# A Draft Genome Sequence of the Burrowing Nematode *Radopholus similis*

**DOI:** 10.21307/jofnem-2019-051

**Published:** 2019-09-17

**Authors:** Reny Mathew, Mark Burke, Charles H. Opperman

**Affiliations:** 1 Department of Entomology and Plant Pathology, North Carolina State University, Raleigh, NC 27695; 2 Accelerated Technologies Laboratories, Inc., West End, NC

**Keywords:** Burrowing nematode, Genomics, Radopholus similis

## Abstract

*Radopholus similis* also known as the burrowing nematode is a devastating pest of banana (*Musa spp.*) and many economically important crops and ornamentals. In this publication, we present the genome assembly of *R. similis*.

Plant-parasitic nematodes cause estimated crop losses of $100 billion worldwide (Coyne et al., 2018). In the United States alone, economic losses due to nematode induced damages have been estimated to be around $10 billion (Coyne et al., 2018). Recent advances in genome sequencing technologies combined with the suite of open source softwares have opened a new paradigm for molecular scientists and plant pathologists to examine the genomic variability that exists within pathogen populations. Starting from the free-living nematode *C. elegans* to the recently published genome of the root-knot nematode *Meloidogyne graminicola* (Somvanshi et al., 2018), nematode genomes and transcriptomes are being heavily explored to understand the underlying complexities that distinguishes a parasitic nematode from their free-living counterparts. The burrowing nematode *Radopholus similis* is a devastating pathogen of banana (*Musa spp.)*, *Citrus* spp., anthurium, black pepper, and numerous other economically important crops and ornamentals (Jacob et al., 2008). Management strategies for *R. similis* are limited and expensive, emphasizing the need to understand the genomic and genetic makeup of this organism in order to seek better and specific tools to combat this parasite.

We sequenced the *R. similis* genome utilizing the Roche 454 platform and assembled it into 8,133 scaffolds with a 30X coverage using Newbler, resulting in an N50 of 13.4 Kb. At ~65 Mbp, the *R. similis* genome assembly size is smaller than the free-living nematode *C. elegans* (~100 Mbp) and multiple plant-parasitic nematodes in clade IV such as *D. destructor* (~112 Mbp) (Zheng et al., 2016), *G. pallida* (~124.6 Mbp) (Cotton et al., 2014), and *B. xylophillus* (~74.6 Mbp) (Kikuchi et al., 2011), although it is slightly larger than the root-knot nematode *M. hapla* (~53 Mbp) (Opperman et al., 2008). The GC content of the *R. similis* is ~51%, which is higher than most plant-parasitic nematodes in clade IV and in keeping with the observations noted by Jacob et al. (2008). NCBI platforms were utilized to pinpoint contaminations in the genome assembly and following this the resulting contaminated sequences were removed. The completeness of the genome assembly was assessed using Core Eukaryotic Gene Mapping Approach (CEGMA) (Parra et al., 2007) and Benchmarking Universal Single Copy Orthologs (BUSCO) (Simão et al., 2015). CEGMA analysis indicates approximately 221 (89.11%) complete and 230 (92.74%) partial genes (>75% amino acids) in the draft *R. similis* assembly. Utilizing the Eukaryota lineage to run BUSCO, we found 85.2% complete genes in the genome assembly and 4.6% fragmented genes. For gene predictions, MAKER v. 2.31.8 (Cantarel et al., 2008) platform was utilized. MAKER gene predictions were made by integrating predictions from evidence such as *R. similis* ESTs, Uniprot, and Swissprot databases, protein datasets of closely related nematode species as well as the *ab initio* tools SNAP and AUGUSTUS. MAKER predicted 18,564 protein-coding genes in *R. similis.* Furthermore, to assess completeness, we also performed a tblastx of the 7,382 *R. similis* expressed sequence tags (ESTs) extracted from NCBI EST database against the *R. similis* draft genome assembly. We found that 6,960 sequences (~94%) had hits in the *R. similis* draft genome assembly and only 422 sequences did not return hits. We hope that this genome assembly could provide valuable insight into the myriad mechanisms of parasitism utilized by this nematode to sustain as a parasite.

GenBank accession numbers:

The Whole genome shotgun (WGS) sequence has been deposited in NCBI Genbank under the accession number SJFO00000000.

## References

[ref002] Cantarel B. L., Korf I., Robb S. M., Parra G., Ross E., Moore B., Holt C., Alvarado A. S. and Yandell M. 2008 MAKER: an easy-to-use annotation pipeline designed for emerging model organism genomes. Genome Research 18:188–196. 18025269 10.1101/gr.6743907 PMC2134774

[ref003] Cotton J. A., Lilley C. J., Jones L. M., Kikuchi T., Reid A. J., Thorpe P., Tsai I. J., Beasley H., Blok V. and Cock P. J. 2014 The genome and life-stage specific transcriptomes of Globodera pallida elucidate key aspects of plant parasitism by a cyst nematode. Genome Biology 15:R43. 24580726 10.1186/gb-2014-15-3-r43 PMC4054857

[ref004] Coyne D. L., Cortada L., Dalzell J. J., Claudius-Cole A. O., Haukeland S., Luambano N. and Talwana H. 2018 Plant-Parasitic Nematodes and Food Security in Sub-Saharan Africa. Annual Review of Phytopathology 56:381–403. 10.1146/annurev-phyto-080417-045833 PMC7340484 29958072

[ref005] Jacob J., Mitreva M., Vanholme B. and Gheysen G. 2008 Exploring the transcriptome of the burrowing nematode Radopholus similis. Molecular Genetics and Genomics 280:1–17. 18386064 10.1007/s00438-008-0340-7

[ref007] Kikuchi T., Cotton J. A., Dalzell J. J., Hasegawa K., Kanzaki N., McVeigh P., Takanashi T., Tsai I. J., Assefa S. A. and Cock P. J. 2011 Genomic insights into the origin of parasitism in the emerging plant pathogen Bursaphelenchus xylophilus. PLoS Pathogens 7 (9): e1002219. 21909270 10.1371/journal.ppat.1002219 PMC3164644

[ref008] Opperman C. H., Bird D. M., Williamson V. M., Rokhsar D. S., Burke M., Cohn J., Cromer J., Diener S., Gajan J. and Graham S. 2008 Sequence and genetic map of Meloidogyne hapla: a compact nematode genome for plant parasitism. Proceedings of the National Academy of Sciences. 105:14802–14807. 10.1073/pnas.0805946105 PMC2547418 18809916

[ref009] Parra G., Bradnam K. and Korf I. 2007 CEGMA: A pipeline to accurately annotate core genes in eukaryotic genomes. Bioinformatics 23:1061–1067. 17332020 10.1093/bioinformatics/btm071

[ref010] Simão F. A., Waterhouse R. M., Ioannidis P., Kriventseva E. V. and Zdobnov E. M. 2015 BUSCO: Assessing genome assembly and annotation completeness with single-copy orthologs. Bioinformatics 31: 3210–3212. 26059717 10.1093/bioinformatics/btv351

[ref011] Somvanshi V. S., Tathode M., Shukla R. N. and Rao, U. 2018 Nematode genome announcement: a draft genome for rice root-knot Nematode, Meloidogyne graminicola. Journal of Nematology 50:111–113. 30451432 10.21307/jofnem-2018-018 PMC6909324

[ref012] Zheng J., Peng D., Chen L., Liu H., Chen F., Xu M., Ju S., Ruan L. and Sun M. 2016 The Ditylenchus destructor genome provides new insights into the evolution of plant parasitic nematodes. Proceedings of the Royal Society B 283:20160942. 27466450 10.1098/rspb.2016.0942 PMC4971208

